# Measurements of the Impact of 3′ End Sequences on Gene Expression Reveal Wide Range and Sequence Dependent Effects

**DOI:** 10.1371/journal.pcbi.1002934

**Published:** 2013-03-07

**Authors:** Ophir Shalem, Lucas Carey, Danny Zeevi, Eilon Sharon, Leeat Keren, Adina Weinberger, Orna Dahan, Yitzhak Pilpel, Eran Segal

**Affiliations:** 1 Department of Molecular Genetics, Weizmann Institute of Science, Rehovot, Israel; 2 Department of Computer Science and Applied Mathematics, Weizmann Institute of Science, Rehovot, Israel; Rutgers University, United States of America

## Abstract

A full understanding of gene regulation requires an understanding of the contributions that the various regulatory regions have on gene expression. Although it is well established that sequences downstream of the main promoter can affect expression, our understanding of the scale of this effect and how it is encoded in the DNA is limited. Here, to measure the effect of native *S. cerevisiae* 3′ end sequences on expression, we constructed a library of 85 fluorescent reporter strains that differ only in their 3′ end region. Notably, despite being driven by the same strong promoter, our library spans a continuous twelve-fold range of expression values. These measurements correlate with endogenous mRNA levels, suggesting that the 3′ end contributes to constitutive differences in mRNA levels. We used deep sequencing to map the 3′UTR ends of our strains and show that determination of polyadenylation sites is intrinsic to the local 3′ end sequence. Polyadenylation mapping was followed by sequence analysis, we found that increased A/T content upstream of the main polyadenylation site correlates with higher expression, both in the library and genome-wide, suggesting that native genes differ by the encoded efficiency of 3′ end processing. Finally, we use single cells fluorescence measurements, in different promoter activation levels, to show that 3′ end sequences modulate protein expression dynamics differently than promoters, by predominantly affecting the size of protein production bursts as opposed to the frequency at which these bursts occur. Altogether, our results lead to a more complete understanding of gene regulation by demonstrating that 3′ end regions have a unique and sequence dependent effect on gene expression.

## Author Summary

A basic question in gene expression is the relative contribution of different regulatory layers and genomic regions to the differences in protein levels. In this work we concentrated on the effect of 3′ end sequences. For this, we constructed a library of yeast strains that differ only by a native 3′ end region integrated downstream to a reported gene driven by a constant inducible promoter. Thus we could attribute all differences in reporter expression between the strains to the different 3′ end sequences. Interestingly, we found that despite being driven by the same strong, inducible promoter, our library spanned a wide and continuous range of expression levels of more than twelve-fold. As these measurements represent the sole effect of the 3′ end region, we quantify the contribution of these sequences to the variance in mRNA levels by comparing our measurements to endogenous mRNA levels. We follow by sequence analysis to find a simple sequence signature that correlates with expression. In addition, single cell analysis reveals distinct noise dynamics of 3′ end mediated differences in expression compared to different levels of promoter activation leading to a more complete understanding of gene expression which also incorporates the effect of these regions.

## Introduction

Studies aimed at understanding the determinants of gene expression have traditionally been focused on promoter and enhancer sequences. However, regulatory information is also encoded in other genomic regions such as the 5′ and 3′ untranslated regions (UTRs) and may even be embedded within the coding regions themselves [1]–[3]. Since measurements of endogenous expression levels of mRNAs [4]–[9] and proteins [10]–[12] represent the net effect of all regulatory regions and regulatory layers (e.g., transcription, translation and mRNA/protein degradation), it is difficult to use such data to dissect the relative contribution of any single genomic region to the overall measured levels. Thus, if the expression level of one gene is higher than another, we cannot tell which regulatory region or combination thereof causes this behavior. The situation becomes even more complicated when considering the recent observations that suggest that the different regulatory layers often affect each other [13]–[22]. In the context of transcription initiation, the challenge of deciphering the regulatory code that maps sequence into expression levels was addressed by separately fusing the promoter of different genes to a fluorescent protein reporter, integrating the resulting constructs into the same genomic location, and then comparing the levels of the reporters for different promoters [23]–[31]. Since strains for different genes in such synthetic libraries differ only in the promoter sequence that is fused to the reporter, this approach allows a direct measurement of the effect of each promoter sequence on gene expression providing important insights into cis-regulatory mechanisms and principles of promoter activation.

Here we adopted this approach to study the independent effect of 3′ end regions on gene expression. Sequences downstream to the promoter are well known to affect expression, yet our knowledge of this effect is usually based on studies that examined single regulatory interactions in the 3′ UTR [32]–[35]. A genome wide view of the interaction network between RNA binding proteins (RBP) and their target mRNA was done in yeast [36], [37] revealing a rich and multidimensional network of interactions. While these results suggest extensive regulation, very few of these interactions were actually shown to affect protein levels.

A systematic comparison of the effect of native 3′ end regions on protein expression, independent of genomic context, in similar ways with which promoter sequences were studied, has not been performed. Thus, basic questions such as what is the range of expression differences due to native 3′ end regions, and what fraction of genes have a 3′ end region that causes a significant effect on expression, are largely open. And our understanding of the sequence determinants, that affect protein expression in 3′ end regions, is limited. In addition, given that protein expression is known to occur in bursts [38], it is interesting to test whether different regulatory layers will affect the dynamics of such protein production bursts differentially.

To study the effect of 3′ end sequences on protein expression we constructed a library of yeast strains that differ only in the 3′ end sequence integrated immediately downstream to a reporter gene (YFP) with a constant promoter. The yeast *S. cerevisiae* lacks RNAi activity [39] and thus serves as a good model system to study more basic mechanisms by which 3′ end sequences modulate protein levels. We measured the effect on expression of 85 different 3′ end constructs, taken from metabolic and ribosomal protein yeast genes. Measuring florescence of the various strains in the library, both in batch and in single cells, we found a continuous and wide span of expression values displaying distinct dynamics. We found that nucleotide composition upstream to the polyadenylation site correlates with expression, highlighting the importance of this genomic region to protein levels and suggesting that the efficiency of 3′ end formation may be partly responsible for the observed sequence-specific difference in expression in our library. Further characterization of these sequence features will be needed to identify the exact mechanisms through which 3′ end sequences affect expression levels to pave the way to a more complete understanding of gene regulation that also incorporates the effect of these regions.

## Results

### Construction of a library of 3′ end reporter strains

**Figure 1:**
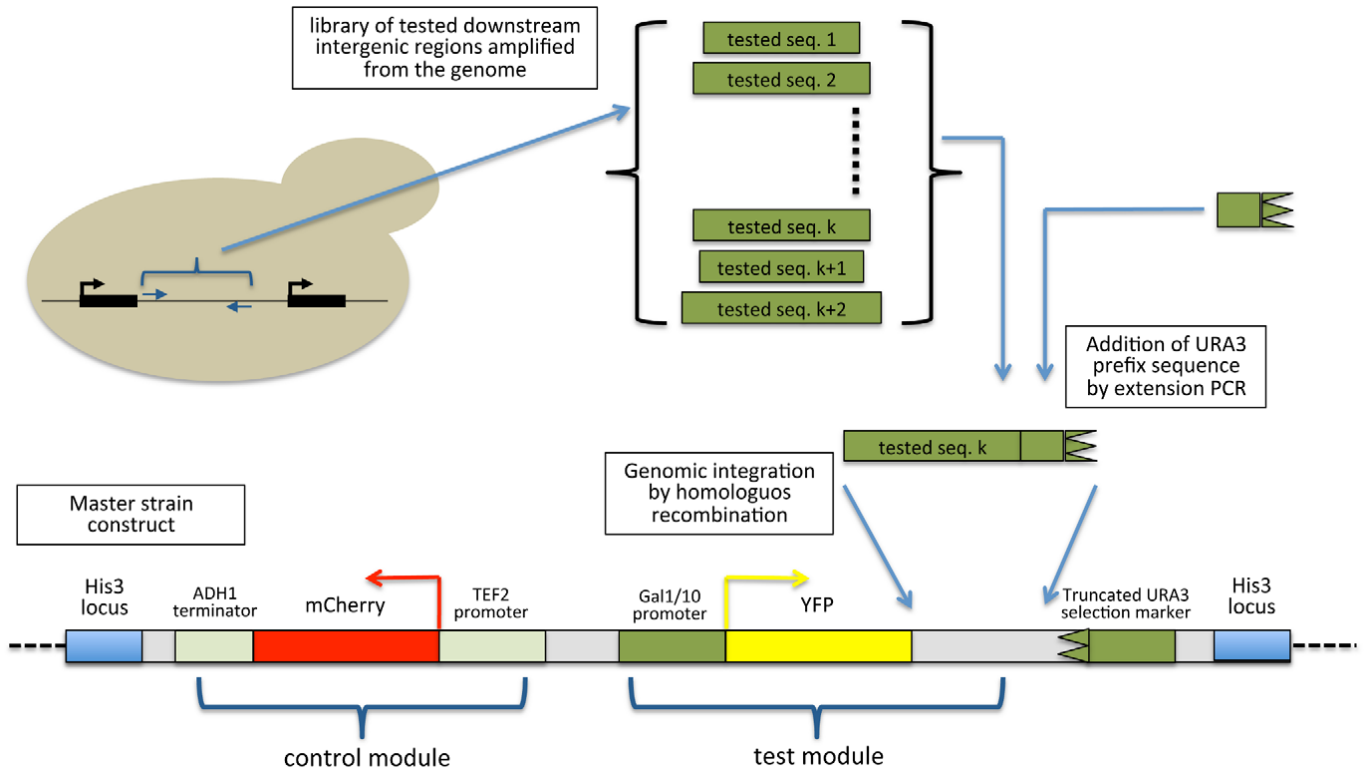
Illustration of the master strain and library construction procedure. A master strain was constructed such that it will contain two main constructs in the HIS deletion locus: a constant control construct with mCherry driven by the TEF2 promoter and terminated by a constant ADH1 terminator; and a test construct with a YFP gene driven by Gal1/10 promoter. Following master strain construction, a library of PCR products containing the downstream intergenic regions of 85 tested genes was amplified from the genome by PCR and extended to also contain the URA3 promoter and start codon. This library of DNA sequences was then integrated into the master strain such that only integrations in the exact genomic location would result in an intact selection marker.

To measure the effect of 3′ end sequences on expression levels, we adapted an approach that we previously developed for measuring the transcriptional effect of promoter regions [29], [31](**Fig. 1**). To this end, we engineered a single master strain to have a genomically integrated YFP reporter downstream to the Gal1/10 inducible promoter [40]–[42], and at the same genomic location, we also integrated an additional fluorescent reporter gene (mCherry) driven by the promoter of TEF2, a transcriptional elongation factor, to be used as a control. A truncated URA3 selection marker, lacking a promoter and start codon, was integrated downstream to the YFP gene to increase specificity of the subsequent transformations. Next, we used PCR to amplify 85 3′ end regions from 47 ribosomal protein genes and 38 galactose metabolism genes from the yeast genome. We attached each selected region to the promoter and start codon of the URA3 selection marker using extension PCR, and separately integrated each of the resulting constructs into the master strain downstream to the YFP reporter using a robotically automated system to increase throughput. Ideally, our constructs should contain only native 3′ UTR sequence, yet the boundaries of the 3′ UTR are not easily defined and most genes show large heterogeneity in the 3′UTR end [6], [43]. In addition, it is not clear how much sequence downstream to the polyadenylation site is required for proper 3′ end formation and transcription termination [44]. For these reasons, we took for each construct the genomic region from the stop codon of the gene of interest to the next open reading frame (up to 1 kb, 12 constructs), while validating that all of the known polyadenylation sites for the gene reside within this region [43]. While it is reasonable to assume that most of the observed effect of each construct is due to the transcribed 3′ UTR sequence, we cannot exclude the possibility that some of the effect is due to sequence elements residing downstream to the 3′ UTR cleavage site, such as interference from a downstream promoter and antisense transcription. To investigate the possibility that the downstream promoter is a major determinant of 3′ end regulated expression, we compared the expression levels of strains containing convergent and tandem intragenic regions (**Fig. S1A**). We did not find any significant difference between the two groups, suggesting that this is not a major contributor to the observed effect. We also checked the effect of the size of the intergenic region and the expression of the downstream gene in both YPD and galactose and found no significant correlation (Fig. S1B–D).

We validated the resulting clones in multiple ways. First, since the same constant promoter drives the mCherry reporter across all strains, mCherry expression should be highly similar across strains, and we thus used it to filter out clones with global transcriptional and growth deficiencies. Second, we used colony PCR and sequencing to validate the integrity of the integrated constructs. Finally, for each construct, we measured the expression of three different clones, and only took clones for which at least two independent strain constructions yielded highly similar YFP measurements (**Fig. S2**). Thus, differences in YFP levels across strains are solely attributable to differences in the 3′ end integrated construct, since the YFP transcripts generated from all library strains are otherwise identical and are all driven by the same Gal1/10 promoter.

**Figure 2:**
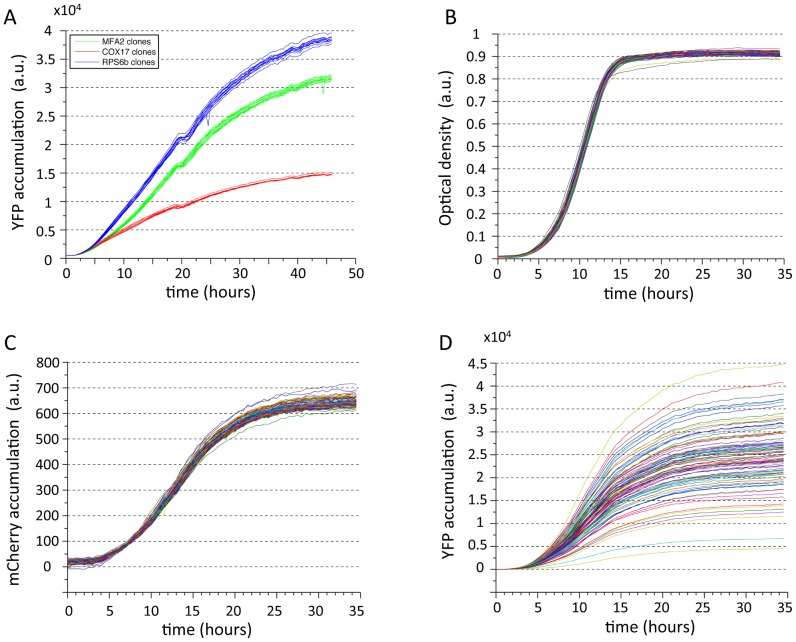
Effect of the 3′ end sequences on YFP accumulation in batch measurements. (**A**) YFP measurements of clones with three different 3′ end sequences. Shown are YFP measurements of three different strains, each with a unique 3′ end sequence. Lines of the same color represent measurements of different clones from the same type of 3′ end sequence, demonstrating that the effect of the different constructs on expression is above the variability of our experimental system. The lowest expressing strain (red) contains the COX17 3′ end and serves as a positive control for our experimental system. (**B,C,D**) Plate fluorometer measurements over time. Following inoculation of the cells in a fresh media containing 2% galactose, optical density (OD), mCherry and YFP are measured over time (B,C and D respectively). Note that as expected, OD and mCherry measurements remain highly similar between different library strains, while YFP expression varies considerably.

To test whether our experimental system can indeed be used to measure post-transcriptional effects, we cloned the 3′ end of COX17 gene, as its transcript is known to be destabilized by the Puf3p RNA binding protein through interaction with the COX17 3′UTR [45]–[47]. As a reference, we also cloned the 3′ ends of MFA2 and RPS6. We found that the COX17 construct indeed resulted in significantly lower YFP levels compared to clones of MFA2 and RPS6. Further, the YFP accumulation profiles of several different clones for each of these three distinct 3′ UTRs were highly similar to one another (**Fig. 2A**).

### Native 3′ end sequences span a wide and continuous range of expression levels

We first examined the overall effect of the integrated 3′ end sequences on YFP expression. Following inoculation in an inducing media containing galactose we measured the growth (measured as optical density (OD)), mCherry and YFP levels over time using a robotically automated plate fluorometer. As a single value per induction curve, we took the amount of YFP fluorescence produced during the exponential growth phase divided by the integral of the OD curve during the same time period. This results in a measure of the average rate of YFP production per cell per second during exponential phase [29].

**Figure 3:**
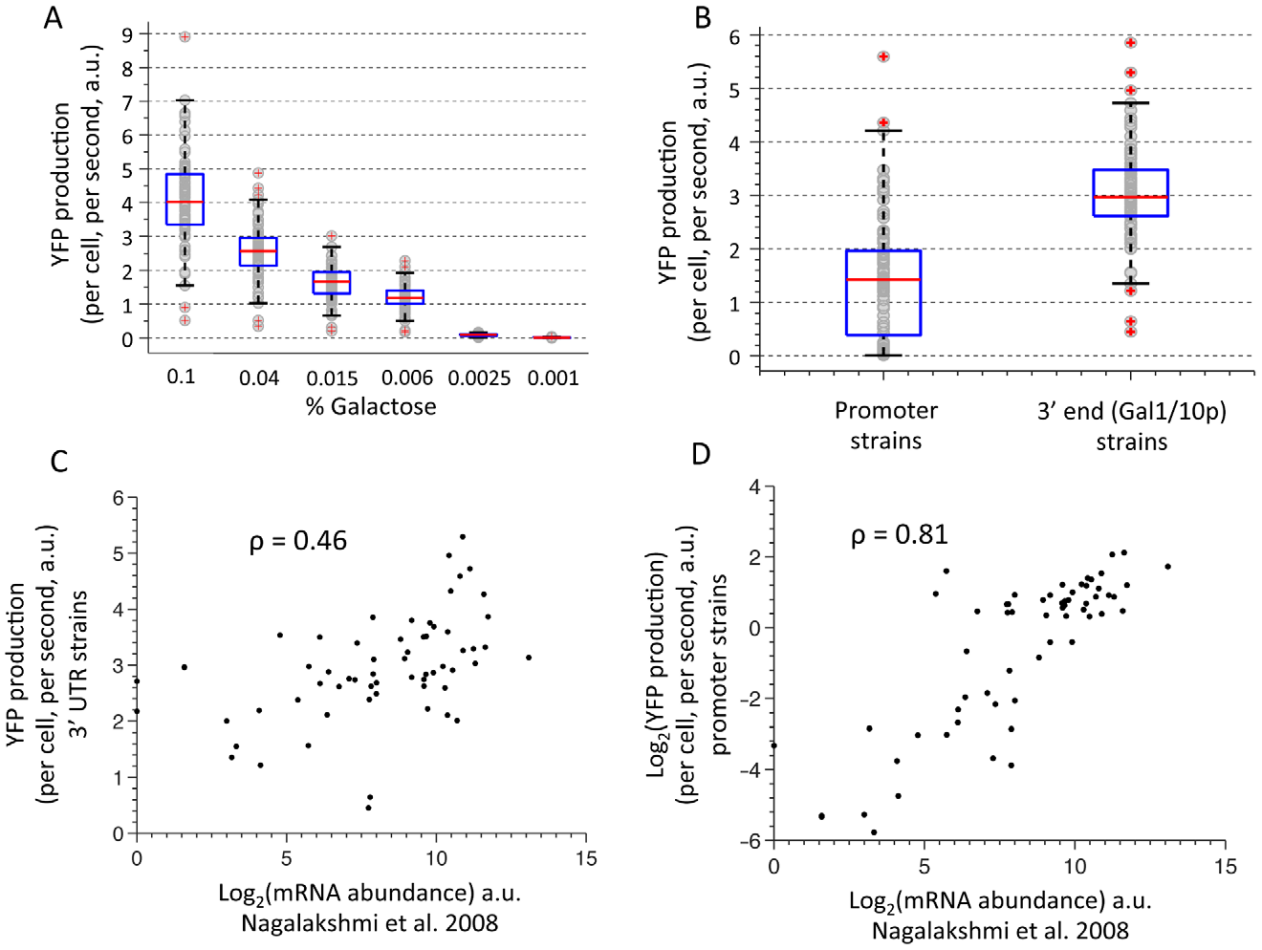
The effect of 3′ end sequences on expression is large and is correlated with endogenous mRNA levels. (**A**) Dynamic range of YFP levels of library strains at different galactose induction levels. YFP production per cell per second was measured and calculated in different Galactose concentrations resulting in different promoter activation levels for all library strains at every galactose concentration. Shown are YFP measurements of the 3′ end library strains. Note that the ratio between the highest and lowest strain at the highest induction level (0.1% galactose) shows a fold difference of more than 10-fold. (**B**) Comparison of the span of expression values between promoter and 3′ end strains for the same group of genes. A box plot is added to show the difference in IQR between the groups. (**C**) Comparison of YFP levels in the 3′ end library (y-axis) with endogenous mRNA levels measured by RNA-seq (x-axis). The Pearson correlation is given (inset). (**D**) Same as (C) but for a different strain library in which promoters of the same respective genes are fused to a YFP reporter.

As expected, the profiles of both OD and mCherry were highly similar across the library strains, such that any measured differences in YFP expression should indeed be attributable to the different 3′ end integrated constructs (**Fig. 2B,C**). Notably, in contrast to the highly similar OD and mCherry measurements across strains, YFP expression levels spanned a wide continuous range of expression values (**Fig. 2D**) across several different Galactose concentrations (**Fig. 3A**). For example, at the highest galactose concentration and thus Gal1/10 promoter induction, we found a 12-fold difference between the YFP levels between the highest and lowest library strains with the lowest expressing strains displaying very low expression levels despite being controlled by a strong Gal1/10 promoter at a highly inducing galactose concentration. To determine if the expression differences between 3′ end regions depends on the specific growth condition or is constitutive and will be observed in other growth conditions we measured the library in a few other conditions and found similar ranking and dynamic range (**Fig. S3**). Our measured differences in florescence between the 3′ end strains may reflect differences in mRNA levels, translation rates, or both. To distinguish between these possibilities, we selected 11 strains from the library that span the whole range of YFP expression values and determined their YFP and mCherry mRNA levels by quantitative PCR (qPCR) (**Fig. S4**). We found very high correspondence between YFP protein and mRNA levels, indicating that most of the observed effect is due to differences in mRNA levels.

A basic open question in gene expression is the quantitative contribution of each regulatory region to the endogenous differences in expression level between genes in their native context. To address this question, we first examined the regulatory potential of the 3′ end sequences by comparing the span of expression values between the 3′ end strains to the span of expression values by a library in which the promoters of the same set of genes were integrated upstream of the YFP reporter [29] (and unpublished data) with a constant 3′ UTR. The 3′ end strains exhibit approximately half of the dynamic range as the promoter strains (interquartile range of 0.84 and 1.5 for 3′ end and promoters, respectively) (**Fig. 3B**). This range is observed at the highest galactose induction level and thus represents the maximal regulatory potential of these sequences and not their endogenous effect under lower promoter expression levels, as the range of effect of the 3′end sequences scales with promoter expression. Next, to see how much of our measurements explain endogenous variability in mRNA levels, we compared the YFP expression of each 3′ end library strain to mRNA levels of the respective genes in the native genomic context measured by RNA-seq [6] (**Fig. 3C**). We found the correlation between these two measurements to be relatively high (R = 0.46), especially considering that we measure the effect of only the 3′ end separated from its native genomic region and promoter. As a reference, we also compared the same RNA-seq mRNA level values to measurements of the promoter strains and found, as expected, a higher correlation (**Fig. 3D**, R = 0.81). Combining these two measures in a regression analysis against endogenous mRNA levels [6] modestly increased the amount of explained variance (R^2^) to 71% compared to 66% with only promoters (**Fig. S5**). To test the significance of this increase, considering the additional free parameter in the model containing the 3′ end measurements, we computed the F-statistic for nested regression models and obtained a p-value of 0.002.

### Sequences with higher A/T content upstream to the polyadenylation site have higher expression

Because differences in the YFP expression levels of strains in our library are attributable to differences in the integrated 3′ end sequences, we next sought to identify sequence features that cause the measured differences in YFP levels. Unlike promoter sequences, where many regulatory elements such as transcription factor binding sites and TATA box are known, relatively little is known about regulatory elements or general sequence features in 3′ end, and so far a few putative regulatory motifs have been predicted [1], [47].

Accurate sequence analysis depends on our knowledge of the location of polyadenylation site (3′ UTR end). To determine if our cloned sequences create the same 3′ UTRs as in the native context, we used 3′ RACE followed by deep sequencing (see materials and methods) to map the distribution of polyadenylation sites for each of our strains. We created a high resolution map of polyadenylation sites for the 64 3′ UTRs with a sufficient number of reads (>1000) (**Fig. S6**). We found multiple polyadenylation sites for a large fraction of genes, as previously observed [6], [43], and also much heterogeneity around each site. We compared the main polyadenylation site between our data and a published dataset that mapped genome wide polyadenylation sites [43] and found high correspondence between the two maps (**Fig. S7**). This shows that the formation of 3′ UTRs is intrinsic to the local 3′ end sequence and independent of the higher genomic context. We used the polyadenylation site with the highest number of reads for each gene as the 3′ UTR end for subsequent analysis and the published polyadenylation site mapping [43] for 3′ UTRs for which we did not have sufficient data.

**Figure 4:**
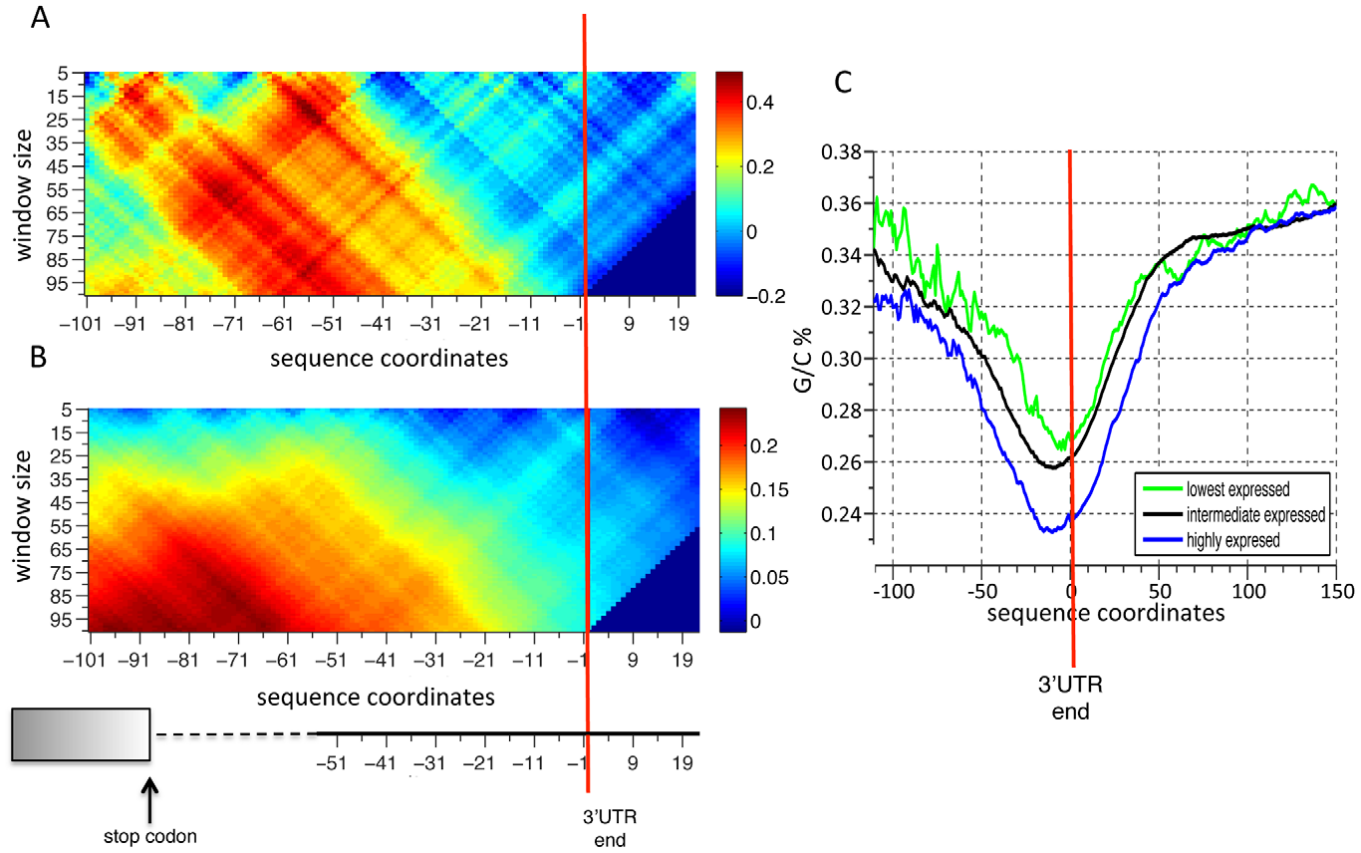
Higher A/T content upstream of the polyadenylation site is associated with higher YFP expression. (**A**) The correlation between A/T content and YFP levels in different window sizes and different locations with respect to the main polyadenylation site. Each point in the matrix represents a different window size (y-axis) centered on a different location (x-axis) with respect to the polyadenylation site. Colors represent the Pearson correlation coefficient (side bar). (**B**) Same as A using genome wide sequence and mRNA levels [6] data. (**C**) Shown is the average G/C content of three sets of genes grouped by their mRNA expression levels (0.2 percentile of the lowest and highest expressing genes and the intermediate group contains all the rest) as a function of the distance from mapped transcription end sites, in windows of 20 bp centered around each point.

We first searched for sequence features (G/C content of the 3′ UTR, enriched k-mers, minimal free energy of the predicted secondary structure of the 3′UTR and 3′UTR length) computed over the whole 3′ UTR, that might explain the measured expression variability, but found no significant correlation between any of these measures and measured YFP levels. We next looked for positional information by aligning our cloned sequences by either the beginning (right after the stop codon) or the end of the 3′ UTR defined as the location of the most common polyadenylation site according to mapped 3′ end locations. We then computed the same features in sliding windows of various size and locations and correlated them with YFP levels to find sequence features that can explain the differential effect on expression of our constructs. Alignment of sequences by the 3′ UTR end revealed that higher A/T content upstream to the 3′ UTR end correlates with higher YFP expression values (**Fig. 4A**). Although higher A/T content may suggest an effect on the stability of the 3′ UTR secondary structure, we found no significant correlation between predicted folding energies and YFP expression. Another possibility, given the proximity of this region in the 3′ UTR to the known location of polyadenylation signals [44], is that increased A/T content results in higher 3′ end formation efficiency which increases protein levels, as has been recently suggested [48]. To determine if the A/T content signal is due to known 3′end processing, we mapped the two most well-known 3′end processing sequence motifs, namely the efficiency and positioning element [44], [49], [50], and found no significant correlation (Fig. S9) between the location of these elements and expression. As the sequence structure of 3′ processing signals is highly variable [51], and given the relatively upstream location of our signal relative to the polyadenylation signal, we hypothesize that increased A/T content at that position creates a more efficient upstream efficiency element (UAS) that enhances 3′ end processing.

To test whether A/T content upstream to 3′ UTR end may indeed be predictive of the effect of 3′ UTRs over all yeast genes, and not just a result of the relatively small number of strains that we used in our analysis, we performed a similar analysis but using all yeast genes and correlating the A/T content of their 3′ UTRs to genome-wide RNA-seq measurements of mRNA abundance [6]. Since endogenous mRNA abundance levels represent the combined effect of all regulatory layers and not just that of 3′ UTRs as in our library, we do not expect a high correlation even if 3′ UTRs contain true regulatory elements that affect mRNA levels. Despite this caveat, we indeed found a low yet highly significant correlation between the A/T content upstream of the 3′ UTR end and mRNA abundance at the genome-wide scale. In contrast to the library this correlation is observed only when A/T content is calculated in larger windows of 70–90 bp (**Fig. 4B,C**). Together, our results suggest significant association between high expression and high A/T content, in the upstream vicinity of the polyadenylation site, and specifically pinpoints the importance of this genomic region to the determination of mRNA and protein levels.

### Different regulatory mechanisms affect protein levels with distinct dynamics

We next asked whether we could gain insights into the way in which 3′ end sequences affect YFP expression. Protein expression occurs in bursts which are characterized by frequency and size, i.e. number of bursts in unit time and number of proteins produced per burst [38], [52]–[54]. We reasoned that an increase in YFP expression can arise from more frequent bursts or from a larger number of proteins produced from each burst, or from a combination thereof. For example, increasing the concentration of a transcription factor is expected to increase more the burst frequency than the size of the burst in the activated promoter state. In contrast, since we expect the effect of 3′ ends on YFP expression to be mediated by changes to YFP 3′ end formation, degradation rates, or by changes to YFP translation rates, we hypothesized that 3′ ends should mainly affect the average size of protein bursts.

Under certain assumptions, burst frequency and burst size can be extracted from single cell protein expression measurements of an isogenic population [54], [55]. We used this approach to measure the effect of 3′ ends on burst frequency and burst size. For comparison, we also modulated transcription activation by varying galactose levels and measured changes in burst size and frequency for each gene across various activation levels. To this end, we used flow cytometry to obtain single cell YFP fluorescence measurements of all of our 85 strains under increasing galactose concentrations, resulting in increasing Gal4 activity [56]. Thus, since all of our library strains are driven by the same target promoter (Gal1/10), comparing the same strain across increasing galactose concentrations allows us to examine the effect of increasing expression by increasing transcription factor activity, while comparing the different strains to each other at a fixed galactose concentration allows us to examine the effect of the various 3′ end regions.

**Figure 5:**
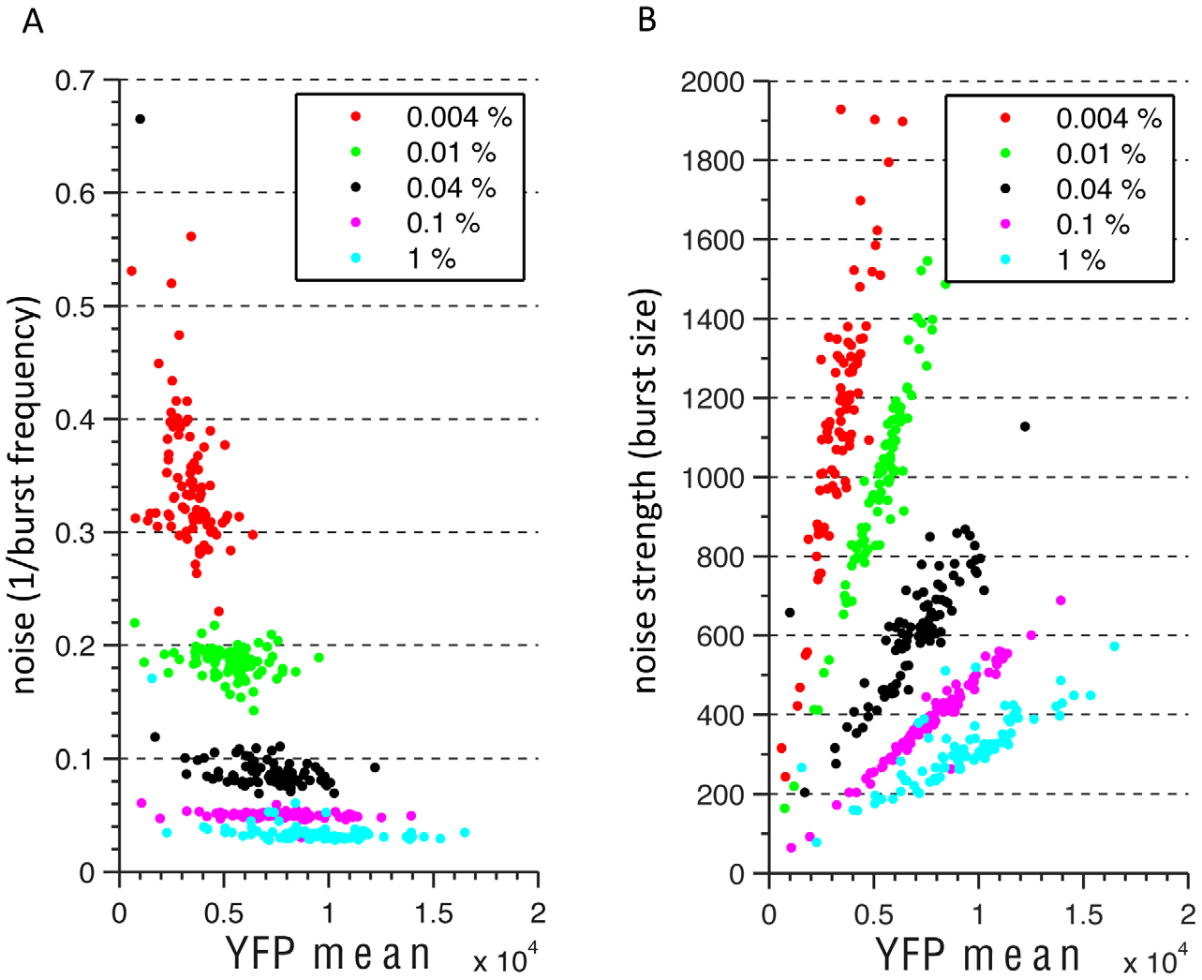
YFP expression is correlated with noise strength. (**A**) For several different galactose concentrations (represented by different colors), shown is the YFP expression of each 3′ end library strain (x-axis) versus its noise (y-axis, expression variance divided by mean expression squared). Each point represents the noise computed from single cell flow cytometry measurements of the corresponding 3′ end strain. (**B**) Same as panel (A) only with noise strength (expression variance divided by mean expression) on the y-axis.

We devised an automated pipeline to extract the mean and variance of a cell population from flow cytometry data, while controlling for variance in physiological cell parameters (see materials and methods). From these measurements, we could then compute the burst frequency of each strain at every galactose concentration as the inverse of the transcriptional noise (variance divided by mean squared), and the burst size as the noise strength (variance divided by mean) [54]. Consistent with our hypothesis, we found that increasing expression by increasing galactose concentration and thus transcription factor concentration results in increased burst frequency, while at any fixed galactose and thus fixed factor concentration, the expression of strains with different 3′ end sequences is correlated with burst size while burst frequency remains constant (**Fig. 5**). We note that while it is appealing to conclude that the expression changes mediated by the different 3′ end sequences have no effect on burst frequency, small changes in such frequencies may not be detected. The differences in burst frequency at different galactose concentrations are mostly driven by fluctuation in the trans activation environment and are probably very large [57], and we can thus only conclude that the effect of our different constructs is very small in comparison. Despite this reservation and even if the assumptions [54], [55] under which our calculations correspond to burst size and frequency do not hold, it is clear that the two different strategies that we examined here for changing expression, namely changing transcription factor activity or changing 3′ end sequences, have vastly different effects on the shape of the distribution of gene expression within an isogenic population.

## Discussion

To assess the quantitative effect of different genomic regions it is essential to establish experimental systems that separate these regions from their native genomic context and measure their direct effect. While it is well established that regulatory features other than the promoter can affect gene expression, to our knowledge our work provides the first systematic measurements of the independent effect of regulatory regions other than promoters in yeast. We show that native 3′ end sequences span a broad and continuous range of expression values of greater than 10-fold. Our library represents a limited number of 85 sequences, chosen without any prior knowledge on their expected effect on expression, and is composed of two unrelated functional groups from the yeast genome. Thus, it is likely that the effect of 3′ end sequences in the genome is larger than the effect we observe due to the small sample size of our library. These genes were chosen as they represent two different regulatory strategies, with ribosomal genes being house-keeping genes expressed constitutively in all growth conditions, and the other group being condition specific genes expressed in the growth condition in which we conduct our measurements. Notably, we did not find any major differences in the 3′end mediated regulation. We quantify the independent effect and explained variance of 3′ end and promoter sequences by comparing our 3′ end library to a promoter library and correlating both to endogenous mRNA levels. The results show that constitutive expression levels are determined by a combination of both regulatory regions. Interestingly, despite the large regulatory potential (dynamic range) of isolated 3′ end constructs on YFP expression, their contribution to the explained variance of endogenous mRNA levels is relatively small. One possible explanation is that the effect of the two regions is not independent; it would thus be interesting to test different 3′ end sequences in different promoter contexts.

Although we cannot say whether the A/T content itself causes higher expression or whether it is a proxy for a more specific signal, our results highlight the 3′UTR end as a genomic region that may have a significant effect on mRNA levels. This sequence signal depends on aligning the sequences by the polyadenylation site. We thus speculate that increased A/T content may result in more efficient 3′ end formation that gives rise to elevated protein expression. It has been previously shown that A/T content is required for efficient 3′ end processing as part of the upstream efficiency element (UAS) [49], [50] [51], [58]. More efficient 3′end processing can result in efficient release of RNA polymerase after polyadenylation and recycling of transcription initiation machinery, given that polyadenylation and transcription termination were shown to be mechanistically coupled [59], [60] [61]–[64]. Additional potential means by which efficient polyadenylation could give rise to higher protein expression comes from a recent work in mammalian cells [48], which suggested that with more efficient 3′ end processing, more transcripts escape from nuclear surveillance, resulting in more mature mRNA molecules exported into the cytoplasm. Notably, all of these mechanisms would result in changes in the size of expression bursts. Although it was shown that by deliberately mutating polyadenylation signals, mRNA and protein levels decrease [65]–[67], we suggest that the efficiency of this process varies between native genes and is partly responsible for the observed variability in protein and mRNA expression in the genome.

Our study demonstrates the strength of a synthetic approach in establishing a causal link between sequence features and their outputs. Observing correlations in the genome, e.g. between sequence features in the 3′ UTR and expression levels could always be explained by indirect non-causal effects. For example, one could argue that the genes with certain UTR features may also have strong promoters. Observing such connections in a setup such as the current library in which the effect of 3′ UTR sequences is measured in isolation partly removes those potential confounders.

Finally, we showed that the observed span of YFP values in our library, mediated by the different 3′ end constructs, affect population noise in a very distinct way compared to expression changes that are mediated by differential promoter activations. Our results thus put 3′ end sequences as appealing candidates for the design of specific circuits in which changes in the mean expression level of a population are needed with little effects on noise. They also demonstrate how the different layers of gene expression regulation affect protein expression with distinct dynamics and propose that such analysis can be used to gain insights into the different layers of regulation involved in an observed change in protein levels.

## Materials and Methods

### Construction of master strain and library strains

The first step in the construction of the 3′ end synthetic library was to build a master strain into which the different 3′ end sequences would be integrated. We built a plasmid containing *TEF2_pr_-*mCherry-*ADH1_term_*, and a non-terminated YFP gene under the control on the Gal1/10 promoter. Downstream of the YFP gene we integrated a truncated URA3 gene lacking the promoter and start codon (**Fig. S8**). The whole construct was then lifted from the plasmid by PCR and integrated into yeast strain Y8205 (courtesy of Charlie Boone) at the *his3* locus (chromosome 15, location 721987–722506 bp). Desired downstream intergenic regions, containing the tested 3′ ends were then lifted from the genome of BY4741 yeast strain. Forward primers had a 3′ end matching the sequence starting immediately downstream to the ORF related to the tested 3′UTR and a 5′ end matching the end of the YFP gene. We planned the library such that the integrated sequences will start from the stop codon till the next ORF (but up to 1 kb if the intergenic region was longer, exact cloned sequences are given in table S1). The reverse primers had a 3′ end matching this location and a 5′ end matching the URA3 promoter lifted from a plasmid with the start codon and a few base pairs into the URA3 promoter to match the sequence in the master strain (**Fig. 1**). Following PCR amplification of the library from the genome the URA3 promoter and start codon were attached to the PCR products using extension PCR and the intact sequences were then integrated into the master strain using homologous recombination to create the library strains. All steps were automated and preformed in a 96 well plate except for the final plating and selection of the final clones which was done in 6 well agar (SCD-URA) plates.

From each transformation 3 clones where manually picked and grown on selective media (SC-URA). Clones for the final library were chosen under the following criteria: (1) At least two clones gave the exact same expression values (**Fig. S2**). (2) integrated sequence length was validated by colony PCR. (3) OD and mCherry measurements were used to ensure no growth of general transcription deficiencies. (4) Few selected inserts were validated by sequencing. Final library strains were grown in 96 well plates containing YPD as a growth medium for two days into stationary phase and frozen by adding glycerol to a final concentration of 25%.

### Acquisition of bulk time course OD and florescence measurements

Cells were inoculated from stocks of −80°C into SC+2% raffinose (180ul, 96 well plate) and left to grow at 30°C for 48 hours, reaching stationary phase. Next, 5ul were passed into a fresh medium (175ul SC+2% raffinose) supplemented with the varying amounts of Galactose. Measurements were carried out every ∼20 minutes using a robotic system (Tecan Freedom EVO) with a plate reader (Tecan Infinite F500). Each measurement included optical density (filter wavelengths 600 nm, bandwidth 10 nm), YFP fluorescence (excitation 500 nm, emission 540 nm, bandwidths 25/25 nm accordingly) and mCherry fluorescence (excitation 570 nm, emission 630 nm, bandwidths 25/35 nm accordingly). Measurements were replicated three times revealing high correlation between independent measurements. We also compared the measurements of the final library plate to the initial measurements of the different clones, representing same strain measured on a different geographical location within the 96-well plate, showing a minimal geographic effect on YFP measures.

### Data processing and calculation of YFP production per cell

We used the same processing pipeline used by Zeevi at al. [29] to quantify promoter activity values from the exact same florescence and optical density measurements. Briefly, background levels are subtracted from OD, mCherry and YFP curves using media measurements, strain with only YFP reporter and strain with only mCherry reporter respectively. Next outlier removal was done on the measurement points which compose each individual curve, removing points which deviate considerably from their neighboring points. We validated by eye that all strains have same OD and mCherry curves (**Fig. 2**). To calculate one value per induction curve an automated procedure divides the OD curve into four growth phases: lag phase, exponential phase, linear phase and stationary phase. Then YFP accumulation was calculated by dividing the total amount of YFP produced during exponential phase by the integral of the OD curve during this time. Because both YFP and mCherry are very stable proteins, this measure represents the amount of YFP or mCherry produced per cell over this time course (table S1). We used YFP measurements not normalized to mCherry, but taking normalized values achieves qualitatively similar results (data not shown). mCherry expression was used for two main purposes: (1) In the clone selection process it allows us to discard strains with growth and global transcription deficiencies. (2) In the noise measurements, gating on mCherry expression was used to minimize the effect of extrinsic factors on YFP expression.

### qPCR analysis for quantification of mRNA levels

Eleven strains spanning the whole range of expression values were randomly selected for quantification of mRNA levels of both YFP and mCherry. Strains were grown in a 96 well plate with 6 replicate wells for each strain in rich media until stationary phase. 5ul of stationary cells were then inoculated into fresh synthetic media (175ul) with 2% galactose to induce expression. Cells were collected after 4.5 hours from mid log phase centrifuged and pellet was immediately frozen in liquid nitrogen. RNA was then extracted using Yeast MasterPure kit (Epicenter Biotechnologies) with a long (1 hour) DNAse treatment to avoid contaminations of genomic DNA. cDNA was prepared using M-MLV reverse transcriptase and random hexamers primers. Quantitative PCR analysis was performed by RT-PCR (StepOnePlus, Applied Biosystems) using ready-mix kit (KAPA, KK4605) with primers spanning the ORF of either YFP (Fw-CCAGAAGGTTATGTTCAA, Rv- CGATTCTATTAACTAAGGTATC) or mCherry (Fw-TGTGGGAGGTGATGTCCAACTTGA, Rv- AGATCAAGCAGAGGCTGAAGCTGA) mRNA molecules in 20 ul volume with triplicate wells for each reaction. Standard curves were prepared by mixing all samples and preparing 4 serial dilutions of 1∶5.

### Mapping the location of polyadenylation sites

The whole library was grown in a 96 well plate containing rich media to stationary phase. 5ul of stationary cells were then inoculated into a second 96 well plate with fresh synthetic media containing 2% of galactose to induce expression. 50ul of cells in mid-log phase were collected after 6 hours from each well into one tube. Cells were then thoroughly mixed, separated to two replicates, centrifuged and pellet was immediately frozen in liquid nitrogen. RNA was then extracted using Yeast MasterPure kit (Epicenter Biotechnologies) with a long (1 hour) DNAse treatment to avoid contaminations of genomic DNA. YFP specific cDNA was prepared for Illumina sequencing using nested 3′RACE [68]. First-strand cDNA was generated from total RNA using M-MLV and a poly(T) primer (GCTCAAGCCACGACGCTCTTCCGATCTNNNNNNNNNNNNTTTTTTTTTTTTTTTTTTVN). YFP cDNA enrichment was performed using a primer (CTCACAATGTTTACATCACTGCTG) complementary to YFP 440 bp downstream of the start codon and a primer (GCTCAAGCCACGACGC) complementary to the priming sequence on the polyT primer. Second round YFP cDNA amplification was performed using a primer (CACGACGCTCTTCCGATCT) complementary to the poly(T) primer and a primer (TGACTGGAGTTCAGACGTGTGCTCTTCCGATCACCCATGGTATTGATG) complementary to 579 bp downstream of the YFP start codon that contains part of the TruSeq Adapter Index11 primer. The final Illumina sequencing library was prepared by PCR using the TruSeq Universal Adapter primer and the TruSeq Adapter Index11 primer.

Raw reads were then processed to extract only the relevant part of the read that would be mapped to the genomic sequence (without the poly(T)) and mapped to a reference genome containing only the cloned sequences using NovoAlign software (http://www.novocraft.com/). The junction between the poly(T) and the mapped genomic sequence was taken as the cleavage site. Our mapping resolution is thus limited to the first non-A nucleotide upstream to the real cleavage site.

### Flow cytometry measurements and extraction of transcriptional burst size and frequency measurements

Similar to the bulk measurements, cells were inoculated from stocks of −80°C into SC+2% raffinose+0.1% of galactose (to induce Gal4p) (180ul, 96 well plate) and left to grow at 30°C for 48 hours, reaching stationary phase. Next, 5ul were passed into a fresh medium (175ul SC+2% raffinose) supplemented with the desired amount of galactose and grown with shaking at 30°C. Six hours following dilution into a fresh medium containing galactose, plates were subjected to flow cytometry measurements using an LSRII flow cytometry machine supplements with an High Throughput Sampler (HTS) to measure 96 well plates.

To control for extrinsic variation we select a sub-population of cells with similar size and physiological status using an automatic gating procedure, which removes cells with spores or with outlier forward and side scatter parameters. Following gating, mean and standard deviation of YFP values was calculated for each strain in each galactose concentration and were used to calculate burst size and frequency following Friedman et al. [54] The general trends presented in this paper were highly robust to changing the gating parameters.

To correct for differences in mean expression between plates measured at different days we used a strain containing a YFP gene driven by the RPL3 promoter which is insensitive to galactose levels. Specifically, for a group of plates the mean expression of the RPL3 promoter across plates was calculated 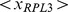. Then the mean expression of each gene in each plate 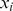 was corrected to be 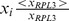 when 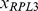 is the mean expression of the RPL3 strain in the specific plate. The noise and noise strength were corrected using the theoretical result that noise^2^ scales with 1/mean such that the noise 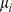 is corrected to be 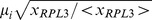 and the noise strength to be the multiplication of the corrected noise and mean. We have verified that this correction does not affect our qualitative results that 3′ end sequences effect mainly noise strength and not noise.

## Supporting Information

Figure S1 **Comparison between convergent and tandem 3′ end constructs.** (**A**) Cumulative distribution of expression values of all library strains. Convergent and tandem 3′ end constructs are colored in black and green respectively showing no significant difference between the two groups. (**B**) YFP expression (y-axis) is plotted against the length of the cloned intergenic region (x-axis). (**C**) YFP expression (y-axis) is plotted against mRNA abundance measurements in rich conditions (x-axis) showing no significant correlation (rho = 0.17 pv = 0.29 for tandem, rho = 0.02 pv = 0.89 for convergent). (**D**) YFP expression (y-axis) is plotted against mRNA galactose induction (x-axis) (rho = −0.04 pv = 0.79 for tandem, rho = 0.036 pv = 0.83 for convergent).
(TIF)

Figure S2 **High reproducibility of our experimental system.** Following the transformation of the 3′UTR sequences into the master strain, we selected three clones for each sequence, and measured their OD, mCherry and YFP values over time during a batch growth experiment. As part of the clone validation process we also compared the YFP levels (after 10 hours) of pairs of clones from the same transformation and selected only pairs of clones with very similar YFP expression (up to 15%) that thus display highly reproducible expression. Red dots mark clones that were not taken for the final library.
(TIF)

Figure S3 **Expression of 3′ end library is highly similar across condition.** Shown is the YFP production per cell per second for several growth conditions (y-axis) against a reference growth condition (SC+2% Galactose). The effect of different 3′UTRs remain highly similar across the tested conditions.
(TIF)

Figure S4 **Comparison between mRNA and protein levels for 11 YFP strains.** qPCR measurements of the ratio between YFP and mCherry mRNA (y-axis) plotted against YFP protein expression values. Because mCherry is expected to be constant between the different strains it is used as a loading control for the qPCR. The error bars represent the standard deviation between 3 technical replicates.
(TIF)

Figure S5 **Regression analysis with and without 3′ end measurements.** Promoter YFP measurements (predictors) were regressed against published endogenous mRNA levels (response variable) with and without the 3′ end YFP measurements using multiple linear regression. The real values (x-axis) are plotted against the predicted values (y-axis) using a model learned on all the data using only the promoter measurements (upper graph) or both promoter and 3′ end (lower graph). The amount of explained variance (inset) increased from 66% to 71%. Because there is an additional free parameter when adding the 3′ end measurements we computed a p-value for the significance of this increase using an F-test for nested regression models resulting in a p-value of 0.002.
(TIF)

Figure S6 **High resolution mapping of cleavage sites for four example genes.** The number of reads (y-axis) for each position downstream to the stop codon (x-axis) is plotted for four representative genes to show the difference between genes in the heterogeneity of cleavage sites.
(TIF)

Figure S7 **Comparison of the main cleavage site between our measurements to published data sets.** The cleavage site covered by the highest number of reads is chosen for both our measurements and published sites (Ozsolak et al. 2010) and plotted once against the other. High correspondence is observed for most genes. The outliers mostly represent cases in which clear multiple sites are observed.
(TIF)

Figure S8 **Map of the plasmid used to construct the master strain.**
(TIF)

Figure S9 **Occurrence of exact matches to known 3′end-processing motifs is not significantly correlated with the expression level of the corresponding 3′ UTR strain.** Sequences are sorted by expression (right panel), each line represents a cloned construct (aligned by its 3′UTR end site) colored according to the G/C content (gray – AT, white – GC) and markings of exact matches to known 3′ motifs (green and red lines mark efficiency and positioning elements, respectively).
(TIF)

Table S1 **Data for each cloned sequence.** Column 1 – systematic name for the gene associated with the 3′end sequence. Column 2 – YFP expression (a.u.). Column 3 – cloned sequence.
(XLS)
