# Supplementary figures and images for: Measurements of the Impact of 3′ End Sequences on Gene Expression Reveal Wide Range and Sequence Dependent Effects
Source: PLoS Comput Biol. 2013 Mar 7;9(3):e1002934. doi: 10.1371/journal.pcbi.1002934 (PMC3591272; doi:10.1371/journal.pcbi.1002934)

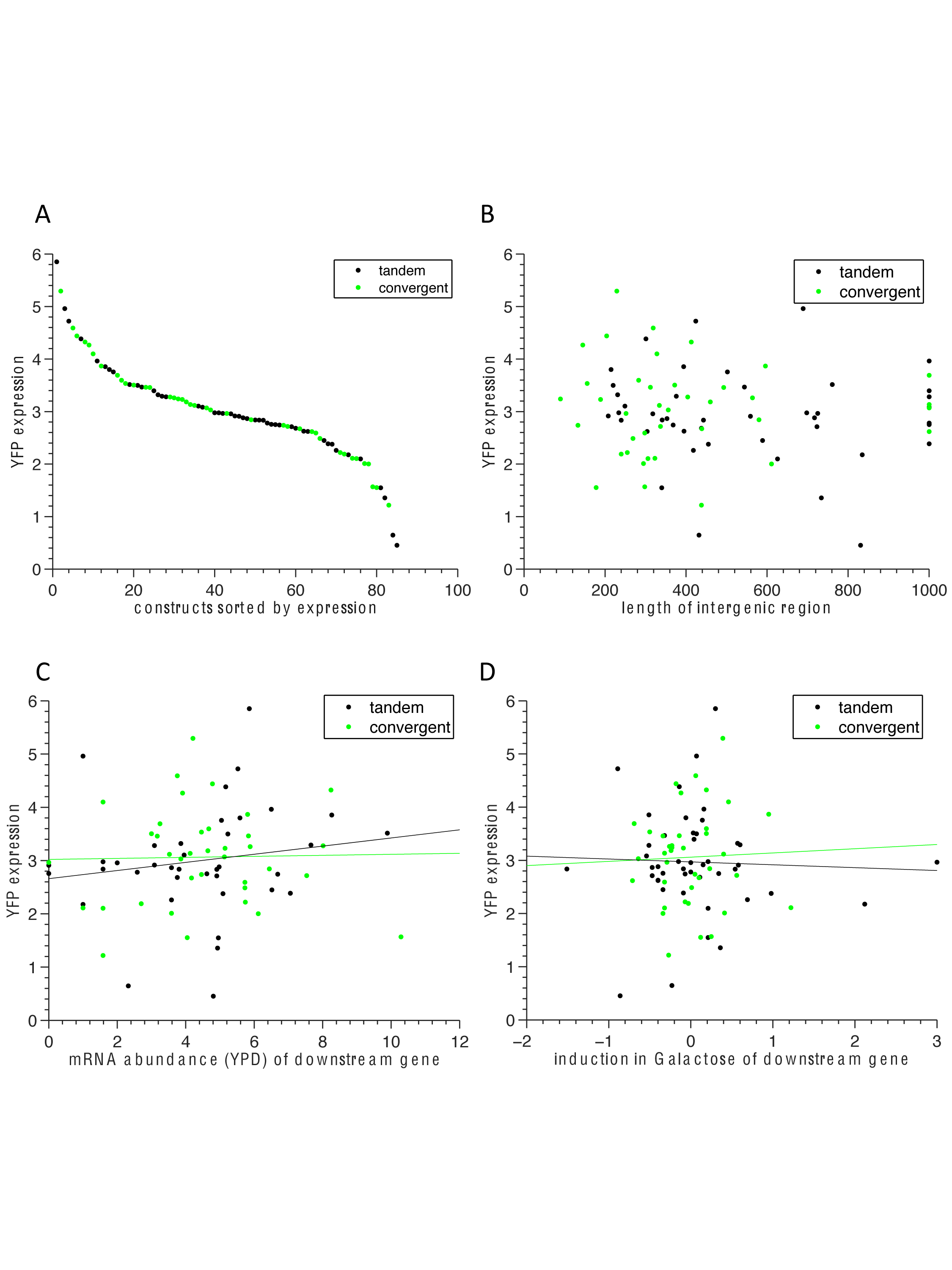

Supplement: Figure S1 — Comparison between convergent and tandem 3′ end constructs. (A) Cumulative distribution of expression values of all library strains. Convergent and tandem 3′ end constructs are colored in black and green respectively showing no significant difference between the two groups. (B) YFP expression (y-axis) is plotted against the length of the cloned intergenic region (x-axis). (C) YFP expression (y-axis) is plotted against mRNA abundance measurements in rich conditions (x-axis) showing no significant correlation (rho = 0.17 pv = 0.29 for tandem, rho = 0.02 pv = 0.89 for convergent). (D) YFP expression (y-axis) is plotted against mRNA galactose induction (x-axis) (rho = −0.04 pv = 0.79 for tandem, rho = 0.036 pv = 0.83 for convergent). (TIF) [file pcbi.1002934.s001.tif]

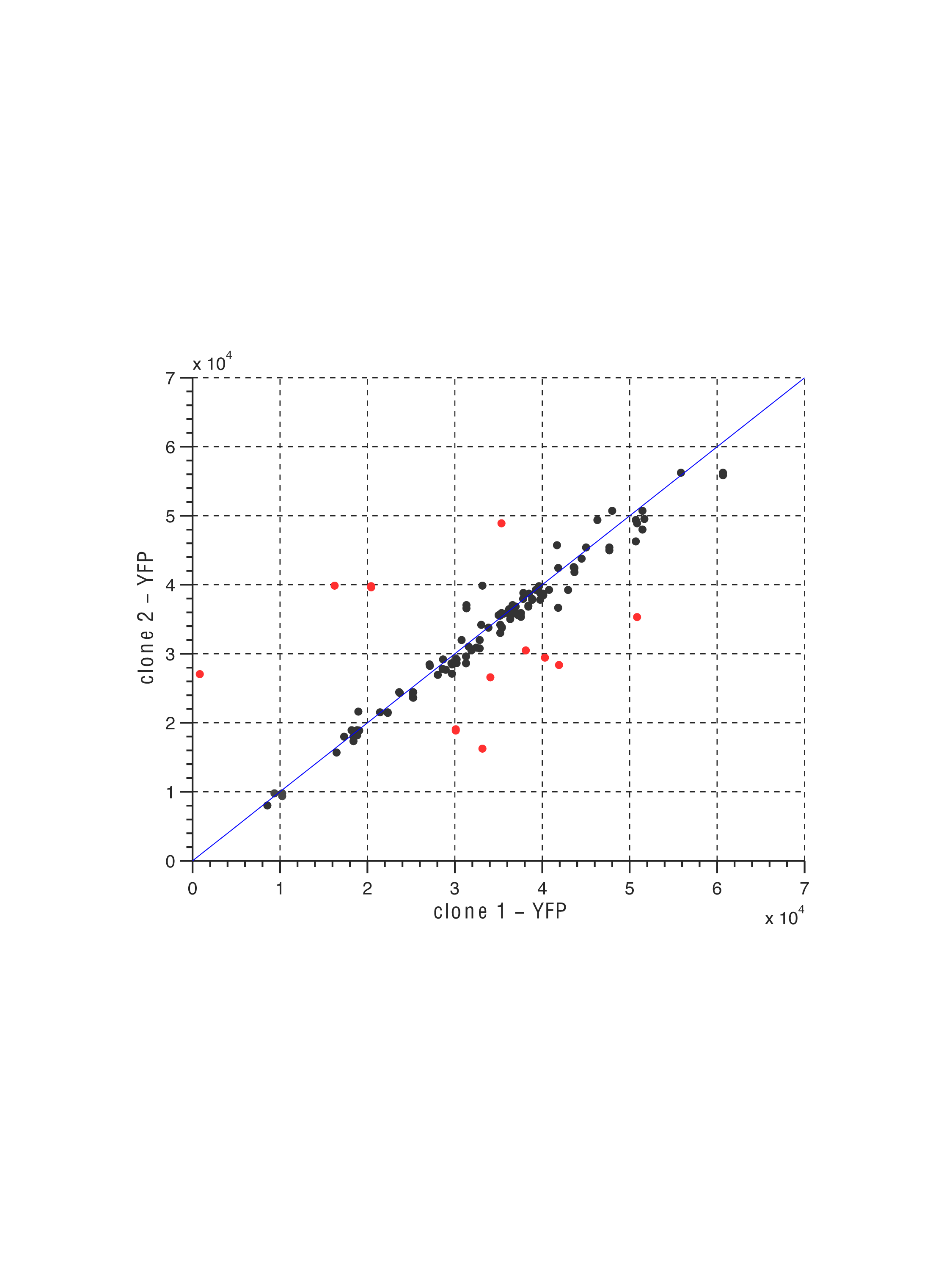

Supplement: Figure S2 — High reproducibility of our experimental system. Following the transformation of the 3′UTR sequences into the master strain, we selected three clones for each sequence, and measured their OD, mCherry and YFP values over time during a batch growth experiment. As part of the clone validation process we also compared the YFP levels (after 10 hours) of pairs of clones from the same transformation and selected only pairs of clones with very similar YFP expression (up to 15%) that thus display highly reproducible expression. Red dots mark clones that were not taken for the final library. (TIF) [file pcbi.1002934.s002.tif]

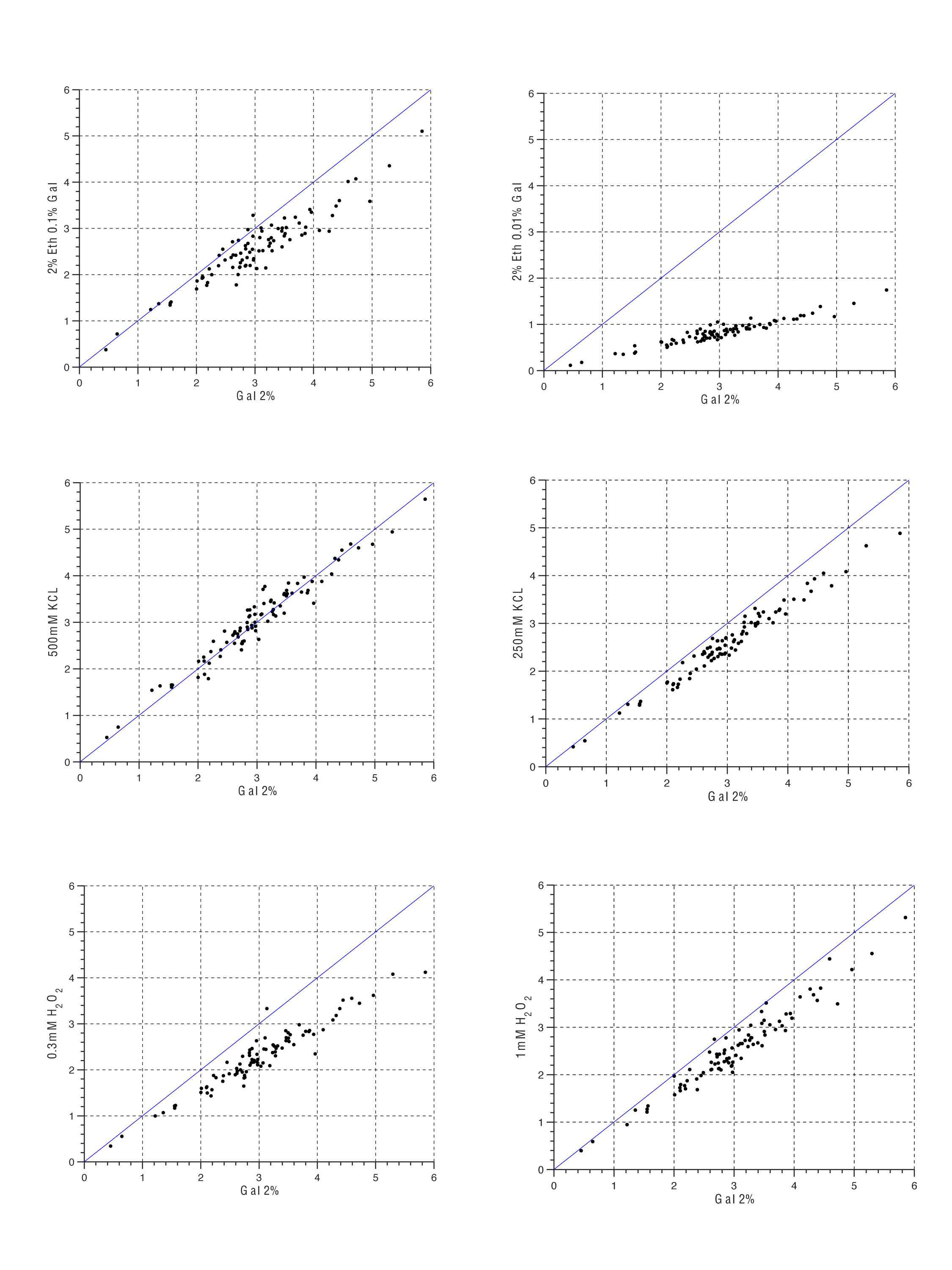

Supplement: Figure S3 — Expression of 3′ end library is highly similar across condition. Shown is the YFP production per cell per second for several growth conditions (y-axis) against a reference growth condition (SC+2% Galactose). The effect of different 3′UTRs remain highly similar across the tested conditions. (TIF) [file pcbi.1002934.s003.tif]

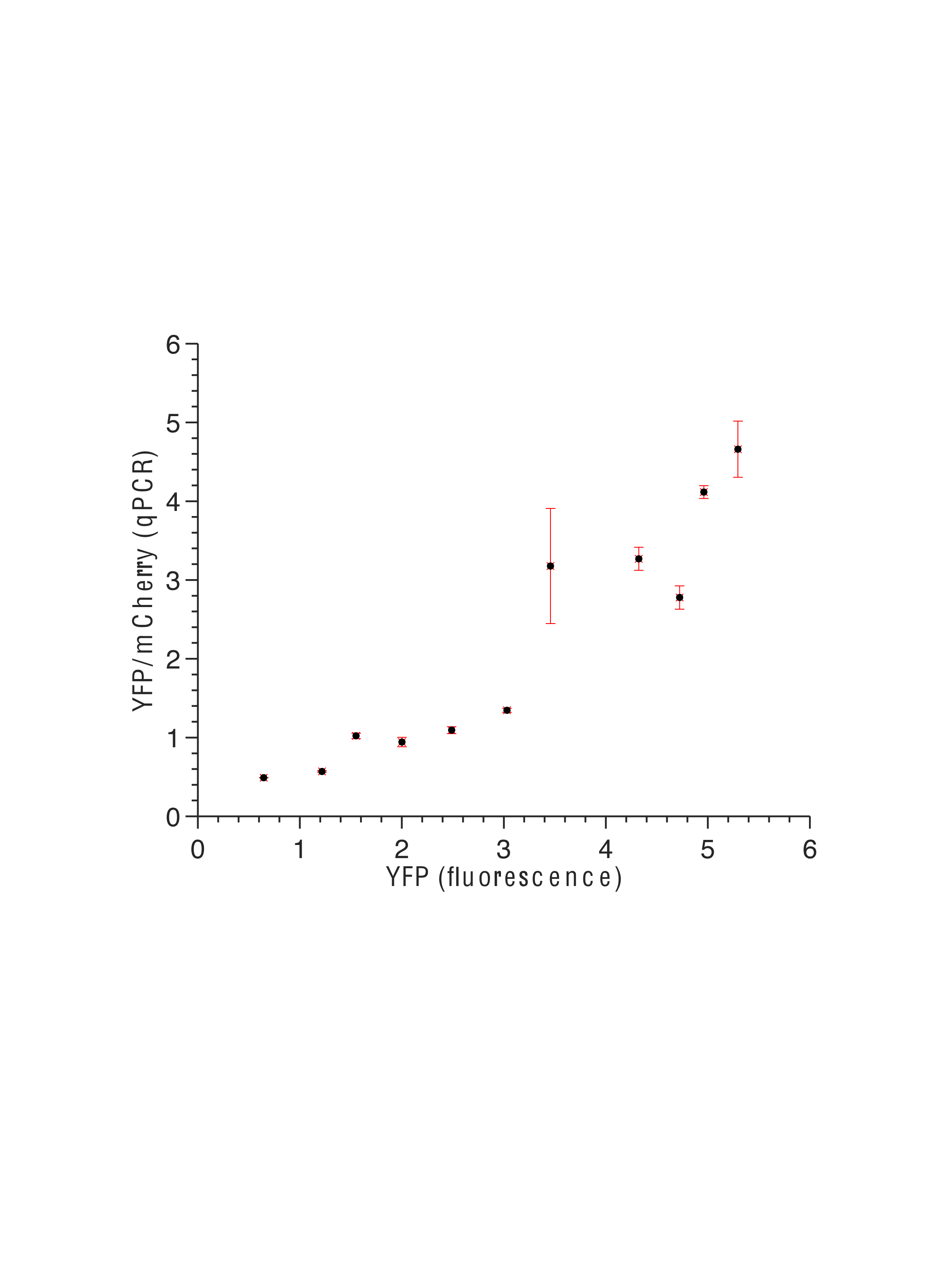

Supplement: Figure S4 — Comparison between mRNA and protein levels for 11 YFP strains. qPCR measurements of the ratio between YFP and mCherry mRNA (y-axis) plotted against YFP protein expression values. Because mCherry is expected to be constant between the different strains it is used as a loading control for the qPCR. The error bars represent the standard deviation between 3 technical replicates. (TIF) [file pcbi.1002934.s004.tif]

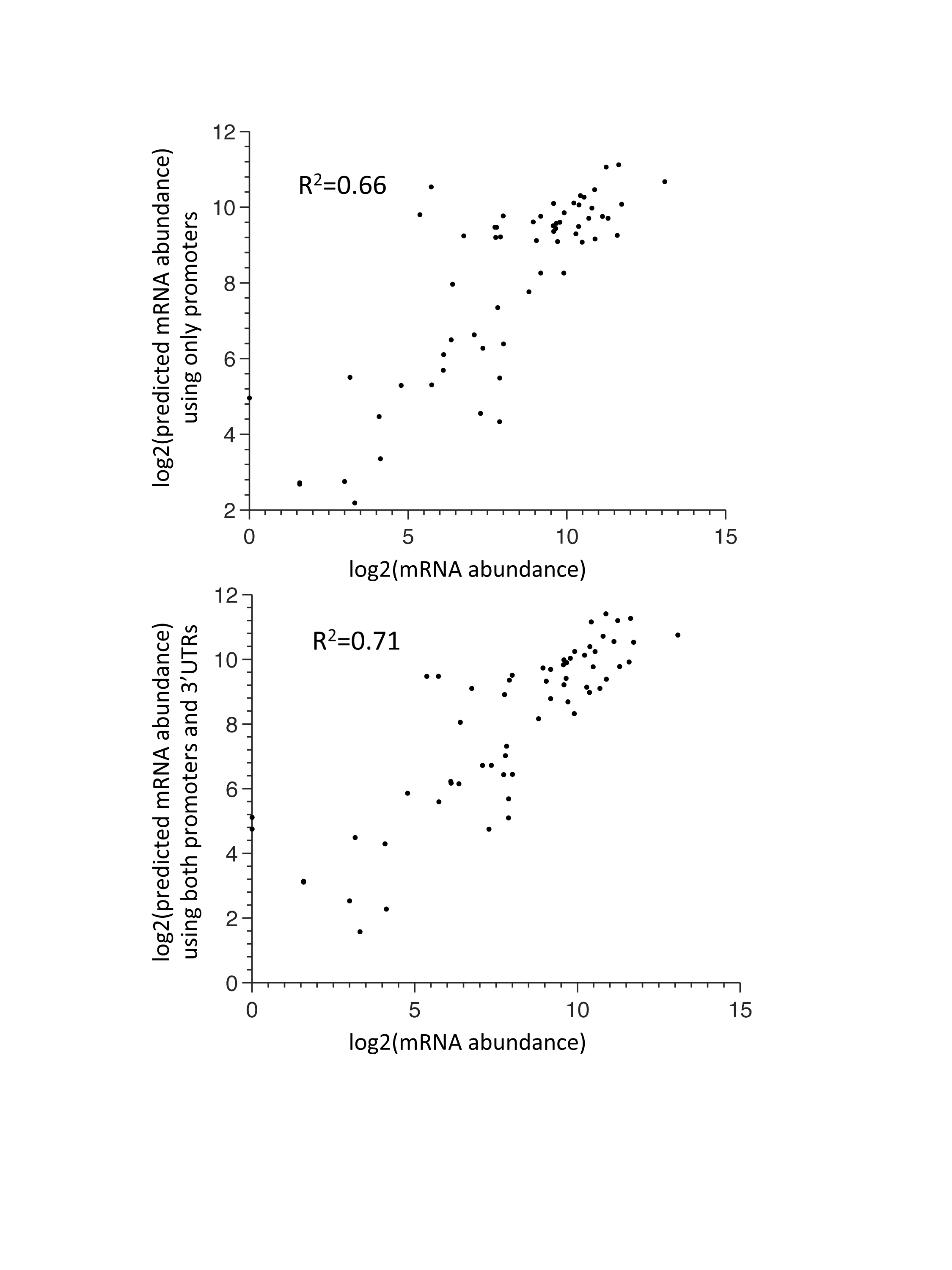

Supplement: Figure S5 — Regression analysis with and without 3′ end measurements. Promoter YFP measurements (predictors) were regressed against published endogenous mRNA levels (response variable) with and without the 3′ end YFP measurements using multiple linear regression. The real values (x-axis) are plotted against the predicted values (y-axis) using a model learned on all the data using only the promoter measurements (upper graph) or both promoter and 3′ end (lower graph). The amount of explained variance (inset) increased from 66% to 71%. Because there is an additional free parameter when adding the 3′ end measurements we computed a p-value for the significance of this increase using an F-test for nested regression models resulting in a p-value of 0.002. (TIF) [file pcbi.1002934.s005.tif]

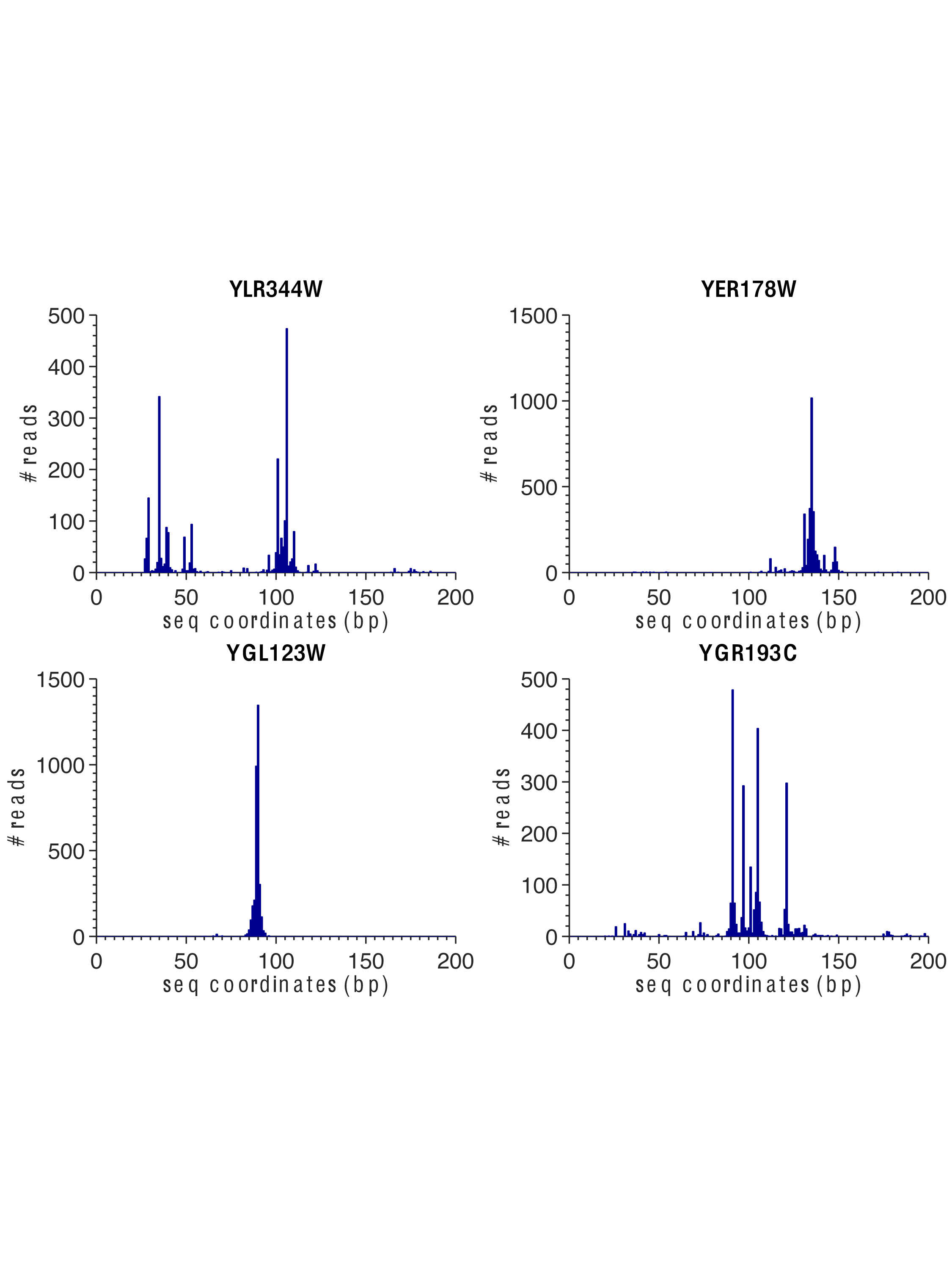

Supplement: Figure S6 — High resolution mapping of cleavage sites for four example genes. The number of reads (y-axis) for each position downstream to the stop codon (x-axis) is plotted for four representative genes to show the difference between genes in the heterogeneity of cleavage sites. (TIF) [file pcbi.1002934.s006.tif]

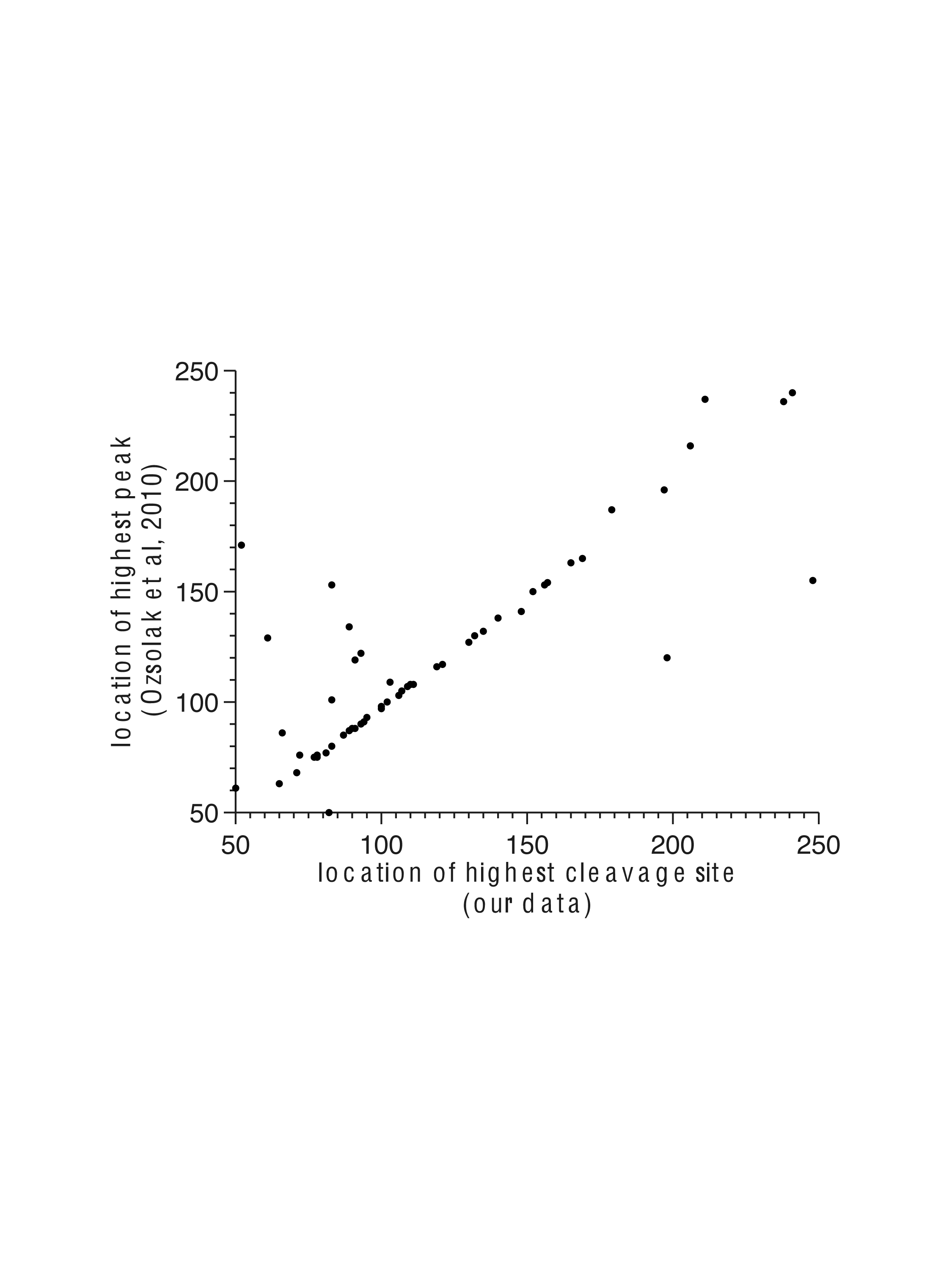

Supplement: Figure S7 — Comparison of the main cleavage site between our measurements to published data sets. The cleavage site covered by the highest number of reads is chosen for both our measurements and published sites (Ozsolak et al. 2010) and plotted once against the other. High correspondence is observed for most genes. The outliers mostly represent cases in which clear multiple sites are observed. (TIF) [file pcbi.1002934.s007.tif]

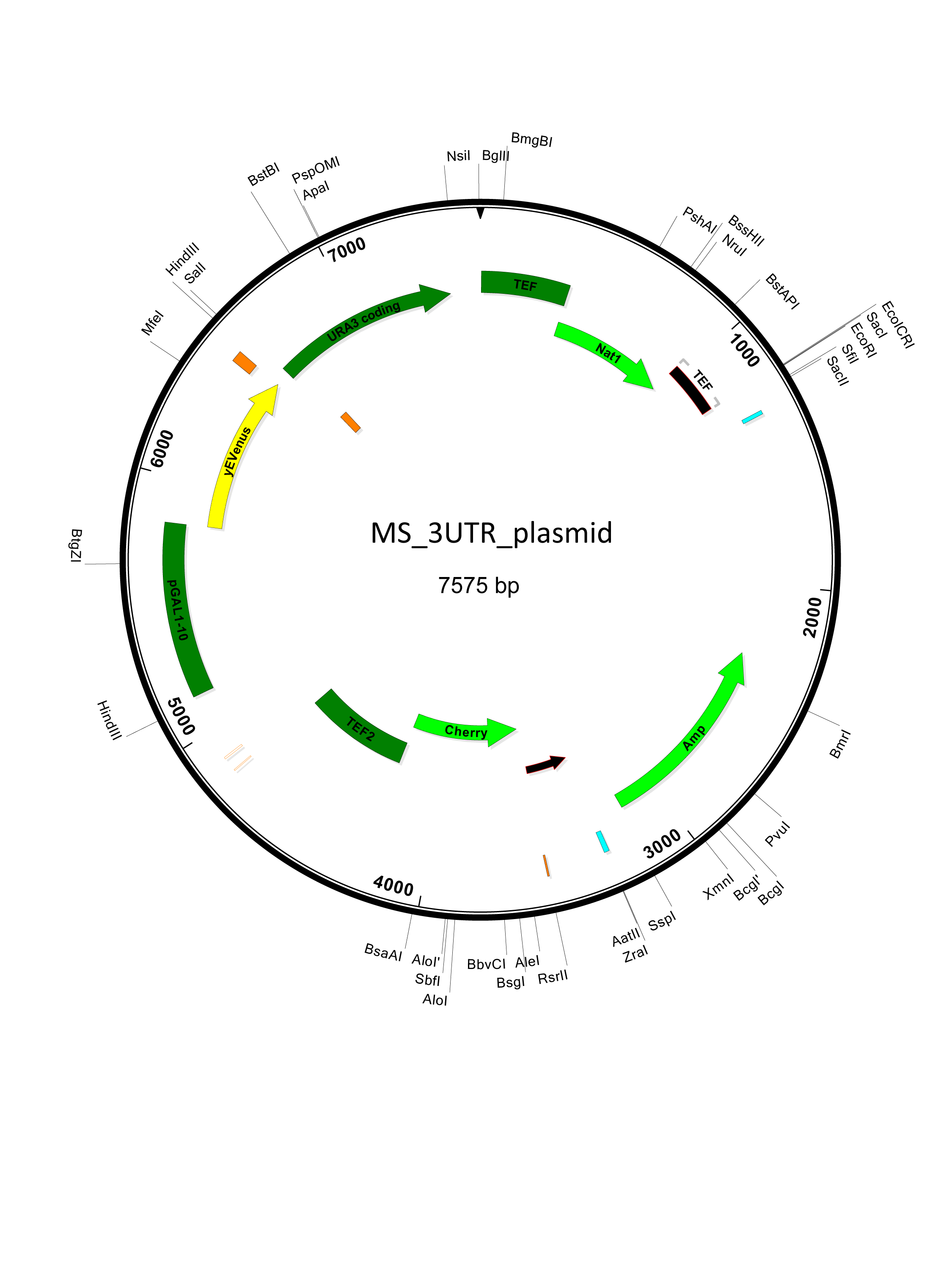

Supplement: Figure S8 — Map of the plasmid used to construct the master strain. (TIF) [file pcbi.1002934.s008.tif]

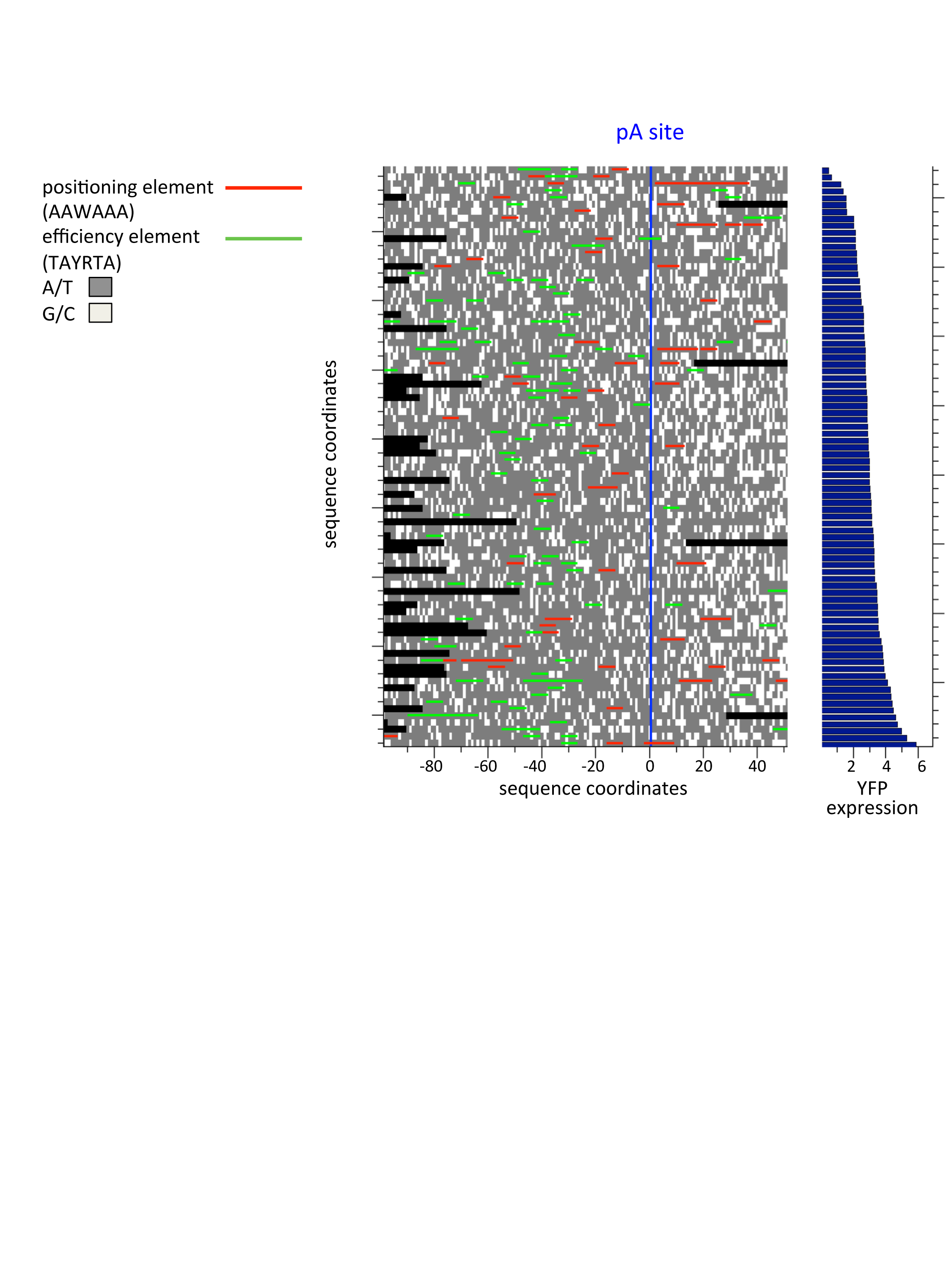

Supplement: Figure S9 — Occurrence of exact matches to known 3′end-processing motifs is not significantly correlated with the expression level of the corresponding 3′ UTR strain. Sequences are sorted by expression (right panel), each line represents a cloned construct (aligned by its 3′UTR end site) colored according to the G/C content (gray – AT, white – GC) and markings of exact matches to known 3′ motifs (green and red lines mark efficiency and positioning elements, respectively). (TIF) [file pcbi.1002934.s009.tif]
